# Tension Sensor Based on Fluorescence Resonance Energy Transfer Reveals Fiber Diameter-Dependent Mechanical Factors During Myelination

**DOI:** 10.3389/fncel.2021.685044

**Published:** 2021-08-02

**Authors:** Takeshi Shimizu, Hideji Murakoshi, Hidetoshi Matsumoto, Kota Ichino, Atsunori Hattori, Shinya Ueno, Akimasa Ishida, Naoki Tajiri, Hideki Hida

**Affiliations:** ^1^Department of Neurophysiology and Brain Science, Nagoya City University Graduate School of Medical Sciences, Nagoya, Japan; ^2^Supportive Center for Brain Research, National Institute for Physiological Sciences, Okazaki, Japan; ^3^Department of Physiological Sciences, The Graduate University for Advanced Studies, Okazaki, Japan; ^4^Department of Materials Science and Engineering, Tokyo Institute of Technology, Meguro, Japan

**Keywords:** oligodendrocyte, myelination, tension sensor, mechanical factor, fluorescence resonance energy transfer

## Abstract

Oligodendrocytes (OLs) form a myelin sheath around neuronal axons to increase conduction velocity of action potential. Although both large and small diameter axons are intermingled in the central nervous system (CNS), the number of myelin wrapping is related to the axon diameter, such that the ratio of the diameter of the axon to that of the entire myelinated-axon unit is optimal for each axon, which is required for exerting higher brain functions. This indicates there are unknown axon diameter-dependent factors that control myelination. We tried to investigate physical factors to clarify the mechanisms underlying axon diameter-dependent myelination. To visualize OL-generating forces during myelination, a tension sensor based on fluorescence resonance energy transfer (FRET) was used. Polystyrene nanofibers with varying diameters similar to neuronal axons were prepared to investigate biophysical factors regulating the OL-axon interactions. We found that higher tension was generated at OL processes contacting larger diameter fibers compared with smaller diameter fibers. Additionally, OLs formed longer focal adhesions (FAs) on larger diameter axons and shorter FAs on smaller diameter axons. These results suggest that OLs respond to the fiber diameter and activate mechanotransduction initiated at FAs, which controls their cytoskeletal organization and myelin formation. This study leads to the novel and interesting idea that physical factors are involved in myelin formation in response to axon diameter.

## Introduction

In the central nervous system (CNS) white matter, large and small caliber axons are intermingled, and the diameter of myelin internodes is highly divergent (Weruaga-Prieto et al., 1996), especially in the spinal cord. Large diameter axons are more suitable for being myelinated than small diameter axons (Friede, 1972). Additionally, the ratio of [axon diameter] to [axon + myelin diameter] (g-ratio) is adjusted to optimum values for each axon. Optimization of the g-ratio is important for higher brain functions. This indicates that myelin formation is tightly associated with the axon caliber, involving unknown diameter-dependent regulatory factors.

A previous study has reported that OLs can myelinate axons of paraformaldehyde-fixed dorsal root ganglion neurons similarly to live axons (Rosenberg et al., 2008). Lee et al. (2012) have previously reported that OL can ensheath a myelin membrane on artificial electrospinning nanofibers without living neurons. These reports indicate that molecular signaling activated by functional proteins on the axonal surface is not required for initiation of myelination, but rather there are permissive axonal cues that initiate myelination (Lee et al., 2012). They also investigated the effect of fiber diameter on myelination using varying nanofiber diameters (0.2–4.0 μm), revealing that larger diameter fibers (more than 0.4 μm) were preferentially ensheathed by OLs. However, the mechanisms underlying optimal myelination according to axon diameter have not been fully elucidated.

Câmara et al. (2009) have previously reported that β1 integrin plays important roles in axoglial interactions that sense axon size and initiate myelination. Reduction in β1 integrin function by its dominant negative form affects myelination of small-diameter axons but not large-diameter axons (Câmara et al., 2009). Integrin is one of the major proteins in the focal adhesion complex. Focal adhesions (FAs) mechanically link the extracellular matrix (ECM) to the cytoplasm and are assemblies for mechanotransduction, which transduce signals from the ECM to the actin stress fibers. Integrin and other FA proteins, such as focal adhesion kinase (FAK), paxillin and talin, play important roles in refining FA complexes in response to mechanical stimulation (Giannone and Sheetz, 2006; Schwartz and DeSimone, 2008; Geiger et al., 2009). Suzuki et al. (2012) have also reported that myelination of small-diameter axons was significantly impaired in the spinal cord of *teneurin-4* deficient mice. Furthermore, Teneurin-4 positively regulated FAK, an essential signaling molecule for myelin formation (Suzuki et al., 2012).

As mentioned above, because integrin is involved in OL-neuron interactions that sense axon size to initiate myelination and is one of the key players in FAs, it is interesting to investigate OL mechanical forces across FAs, which are key platforms for mechanical signal transduction initiated by integrin.

In this study, we thus tried to assess physical OL factors that depend on axon diameter. To visualize the mechanical force generated at OL processes during myelination, we used a previously reported tension sensor (Grashoff et al., 2010). The tension sensor consists of two fluorophores that sandwich a tension sensor module consisting of a 40-amino-acid-long elastic domain (Grashoff et al., 2010). Because fluorescence resonance energy transfer (FRET) enables monitoring protein-protein interactions of two fluorophores, the tension loading on this sensor can reduce FRET efficiency.

We investigated the FRET index of the tension sensor at OL processes contacting nanofibers with different diameters. We found that higher tension was generated at OL processes contacting larger diameter fibers compared with smaller diameter fibers. Additionally, OL formed longer FAs on larger diameter fibers and shorter FAs on smaller diameter fibers. Previous studies have reported that FAs act as mechanotransducers that transmit various signaling pathways (Geiger et al., 2009), which regulate cell morphogenesis and dynamics. These and our results indicate that physical factors are involved in myelin formation in response to axon caliber by activating mechanical signaling initiated at FAs.

## Materials and Methods

### Animals

Neonatal P1 rats of Wistar ST genetic background were purchased from Japan SLC (Shizuoka, Japan). Animal experimental procedures were approved by the Committee of Animal Experimentation of Nagoya City University Medical School and were conducted in accordance with the animal care guidelines of Nagoya City University.

### DNA Construction

The pcDNA3.1-CMV-VinculinTS-mTFP1-mVenus plasmid was a gift from Martin Schwartz (Addgene plasmid # 26019). Super-folder GFP with A206K monomeric mutation (msfGFP) (Zacharias et al., 2002; Pedelacq et al., 2006) was created by introducing mutations by using the QuikChange Site-Directed Mutagenesis kit (Agilent Technologies). The pcDNA3.1-CMV-TSmod-sfGFP/ShadowG plasmid was constructed by inserting msfGFP and ShadowG (Murakoshi et al., 2015) into the pcDNA3.1-CMV-VinculinTS-mTFP1-mVenus plasmid by replacing mTFP1 and mVenus.

### Preparation of Nanofibers

Polymer fibers were prepared by electrospinning from polystyrene (average *M*_w_ ∼ 280,000, Sigma-Aldrich, St. Louis, MO, United States) in solvent mixtures of tetrahydrofuran (THF, Fujifilm Wako, Osaka, Japan) and *N*, *N*-dimethylformamide (DMF, Fujifilm Wako, Osaka, Japan) (the volume ratio of THF and DMF is 1:1). The fluorescent dye, sulforhodamine 101 (Purity > 95.0%, Tokyo Chemical Industry Co., Ltd., Tokyo, Japan), was added to the solutions at a concentration of 0.0025% w/v for fluorescent labeling of fibers. The electrospinning device was the same as that previously described (Matsumoto et al., 2013). For the adjustment of fiber diameter, the solutions with various concentrations were used. Polystyrene fibers with diameters ranging from 0.55 to 4.0 μm were directly electrospun on glass coverslips (18 mm square, thickness of 0.13–0.17 mm, Matsunami Glass Ind., Ltd., Osaka, Japan) from 14 to 22 wt% polystyrene/THF-DMF solutions. The electrospinning conditions were set to keep stable jet formation for each solution: The applied voltage was 12 kV, the flow rate of spinning solution was 0.2–0.5 ml/h; and the distance between the nozzle tip and the collector was 100 mm. The duration of the spinning was 20 sec. The fiber-containing glass coverslips (18 mm square) were put onto the 35 mm culture dishes and applied with elastic adhesive (AX-176, CEMEDINE Co., Ltd., Tokyo Japan) on both edges of the 18 mm coverslips (including both ends of the nanofibers), and then air-dried for at least 24 h. The nanofibers were sterilized for cell culture by placing the fiber-containing glass coverslips under the UV light for 20 min.

### Oligodendrocyte Progenitor Cell (OPC) Culture

The cortices of postnatal day 1 neonatal rats were dissociated and trypsinized, and then cultured on poly-D-lysine (PDL, Sigma, P0899)-coated flasks in DMEM with 10% fetal calf serum (FCS). By 10 days *in vitro*, these cultures consisted of microglia and oligodendrocyte precursor cells growing on an astrocyte monolayer. Microglia were removed based on their differential adhesion to the surface of astrocytes by mechanical shaking at 180 rpm at 37°C for 30 min. Purified OPCs were then acquired by mechanically shaking from the surface of astrocytes at 200 rpm at 37°C overnight. Purified OPCs were seeded on poly-D-lysine (PDL)- or Laminin-coated dishes, and maintained in growth medium: DMEM supplemented with 1 × N2 supplement (Invitrogen, Carlsbad, CA, United States), 0.01% bovine serum albumin, FGF2 (10 ng/ml, R&D systems) and PDGF-AA (10 ng/ml, R&D systems). To induce the differentiation of OPCs, FGF2 and PDGF-AA were removed from the culture medium, and triiodothyronine (T3, 30 ng/ml) was applied. Cells were then maintained in the differentiation medium for 2–5 additional days and fixed with 4% paraformaldehyde.

### Oligodendroglial-Nanofiber Cultures Following Transfection of the Tension Sensor

To coat the nanofiber-containing glass coverslips, we dropped 200 μl of poly-D-lysine solution (final concentration of 0.1 mg/ml) onto an area of the coverslips with nanofibers and incubated for 1 h at room temperature, and coverslips were washed by immersing them by sterile water 3 times and air-dried the coverslips. The fiber-containing glass coverslips were then coated with laminin solution (0.1 mg/ml) for 2 h at 37°C, following the poly-D-lysine coating. One day before transfection, purified OPCs (1.5 × 10^5^ cells per coverslip) were seeded onto the laminin-coated coverslips with nanofibers. Plasmid construct was pcDNA3.1-CMV-TSmod-sfGFP/ShadowG. The cells were transfected with the plasmid using LipofectAMINE 2,000 reagent (Invitrogen). We diluted 2.5 μg of DNA in 50 μl Opti-MEM^TM^ medium (Invitrogen) and 3 μl of LipofectAMINE 2,000 in 50 μl Opti-MEM^TM^ medium (Invitrogen) without serum, and combined the diluted DNA and diluted LipofectAMINE 2,000 (total volume was 100 μl). The transfection complex (100 μl) was applied to each coverslip containing OPCs along with 300 μl of growth medium. After having kept the coverslips containing fibers and OPCs in small volumes of medium for 3 h of incubation, the medium was changed to 2 ml of growth medium and the cells were maintained overnight. On the next day, the medium was changed to 2 ml of myelinating culture medium: composed of 50:50 mixture of DMEM (Invitrogen) supplemented with 1x N2 supplement (Invitrogen) and 0.01% BSA: Neurobasal medium (Invitrogen) supplemented with 1x B27 supplement (Invitrogen) and 1x Gluta-MAX^TM^ (Thermo Fisher Scientific), containing penicillin-streptomycin (Invitrogen), N-acetyl cysteine (5 μg/ml; Sigma) and forskolin (10 μM; Sigma). The medium was changed every 3 days for the remainder of the culture period. The length of the culture period required for optimal FRET observation was 8∼10 days.

### Two-Photon Fluorescence Lifetime Imaging

Details of the 2pFLIM-FRET imaging were described previously (Yasuda et al., 2006). Briefly, msfGFP in the FRET sensor was excited with a Ti-sapphire laser (Mai Tai; Spectra-Physics) tuned to 920 nm. The scanning mirror (6210H; Cambridge Technology) was controlled with a PCI board (PCI-6110; National Instruments) and ScanImage software (Pologruto et al., 2003). The green fluorescence photon signals were collected by an objective lens (60×, 0.9 NA; Olympus) and a photomultiplier tube (H7422-40p; Hamamatsu) placed after a dichroic mirror (565DCLP; Chroma) and emission filter (FF01-510/84 or FF03-510/20; Semrock). Measurement of fluorescence lifetime was carried out using a time-correlated single-photon counting board (SPC-150; Becker & Hickl) controlled with custom software (Yasuda et al., 2006). The construction of fluorescence lifetime image was described previously (Murakoshi, 2021). Briefly, we acquired the mean fluorescence lifetime in each pixel by calculating the mean photon arrival time <*t*> using the following Equation (1).

(1) $$<t>=\int t\,F\,\left(t\right)\,dt÷\int F\,\left(t\right)\,dt-t_{0}$$

where *t*_*o*_ is obtained by fitting the whole image with double exponential functions convolved with an instrument response function as described previously (Murakoshi, 2021). Subsequently, the mean fluorescence lifetime in each pixel was converted to the corresponding color to generate fluorescence lifetime images.

### Evaluation of Fluorescence Lifetime Changes

Fluorescence lifetime changes to the control value were evaluated as follows. The control area was assigned to fluorescent positive OL processes that did not contact nanofibers, and the average fluorescence lifetime value of each pixel at the control area was then calculated for each picture field. If there were several processes that did not contact the nanofibers in a picture field, the fluorescence lifetime of the control value was averaged by those processes to normalize it. The histogram was made by the fluorescence lifetime obtained at each pixel. Color was assigned to the average value of each histogram to construct a fluorescence lifetime image. Since FRET is known to shorten the fluorescence lifetime of the donor fluorophore (Murakoshi, 2021), we measured the fluorescence lifetime changes at fluorescent positive OL processes contacting nanofibers compared to the control area.

### Immunostaining

The cultured cells were fixed with 4% paraformaldehyde (PFA) in 0.1 M phosphate buffer (pH 7.4) and used for immunostaining. Fixed cells were blocked with 5% normal goat serum in phosphate-buffered saline and 0.1% Triton X-100 (PBST) and then incubated with primary antibodies overnight at 4°C. The primary antibodies used were as follows: rat anti-GFP antibody (1/500, Nacalai Tesque, 04404-84), monoclonal anti-α-tubulin antibody (1/500, Sigma, T9026), rat anti-myelin basic protein (MBP) antibody (Millipore, MAB386), monoclonal O4 antibody (1/300, R&D systems, MAB1326) and rabbit anti-paxillin antibody (1/250, abcam, ab32084, clone Y113). After being rinsed with PBST, the cells were incubated with secondary antibodies. The secondary antibodies used were Alexa 488- or 594-conjugated goat anti-mouse, anti-rabbit and anti-rat IgG or goat anti-mouse IgM (Molecular Probes). Fluorescent signals were visualized using AX70 fluorescence microscope (Olympus, Tokyo) and A1Rsi confocal fluorescence microscope (Nikon, Tokyo). The number of OL primary processes was analyzed using “Analyze/Sholl” tool of Fiji software based on ImageJ (NIH).

### Statistical Analyses

All results are expressed as the mean ± SEM. For comparison of two groups, a Student’s *t*-test was used. *p*-values < 0.05 were considered significant. Data for multiple comparisons were analyzed by one-way ANOVA followed by a Tukey-Kramer *post hoc* test using the statistical program GraphPad InStat 3 (GraphPad Software, San Diego, CA, United States). A multiple comparison post-test was performed only if *p* < 0.05. The level of significance was *p* < 0.05.

## Results

### Establishment of a New Assay System to Examine Fiber Diameter-Mediated Mechanical Forces During Myelination Using a Tension Sensor

A previous study has observed that myelinated axons have diameters ranging from 0.3 to 2 μm in the mammalian CNS (Remahl and Hildebrand, 1982). Another report has demonstrated that the threshold for minimum fiber diameter ensheathed by OLs was 0.4 μm (Lee et al., 2012). However, the mechanisms underlying myelination control by axon diameter are not well known.

**FIGURE 1:**
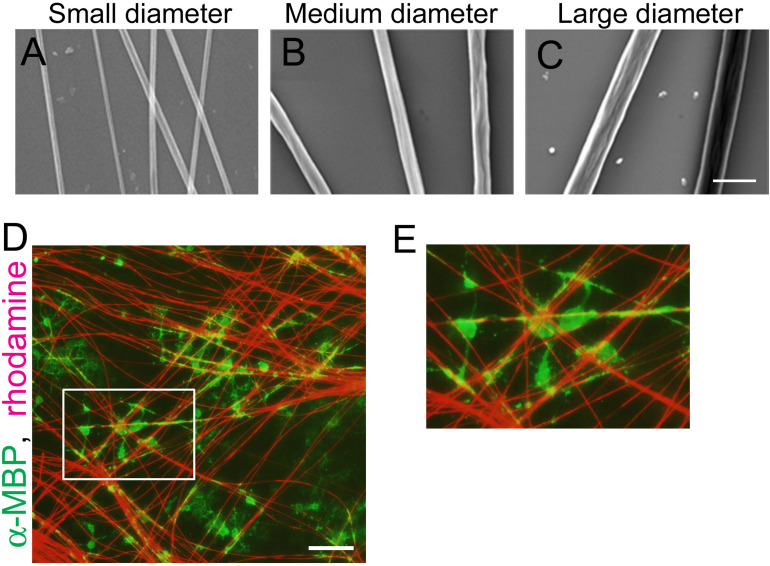
OLs can ensheath polystyrene nanofibers similarly to live axons. **(A–C)** Phase contrast images of polystyrene nanofibers with diameters ranging 0.55–0.9 μm [**(A)**, small diameter], 1.5–1.7 μm [**(B)**, medium diameter] and 2.5–4.0 μm [**(C)**, large diameter]. Scale bar, 5 μm. **(D)** Fluorescent image of MBP (a mature OL marker, green) and sulforhodamine (red), labeling polystyrene nanofibers after 7 days *in vitro*. Scale bar, 100 μm. **(E)** Magnified images of **(D)**, showing OL cells ensheathing nanofibers.

To examine the correlation between axonal fiber diameter and mechanical forces generated at OL processes contacting the fibers, we used polystyrene nanofibers with different diameters. OPCs were seeded on small (0.55–0.9 μm), medium (1.5–1.7 μm), or large-diameter (2.5–4.0 μm) nanofibers (Figures 1A–C). We confirmed that OLs cultured on coverslips with nanofibers maintain their capacity to myelinate the fibers similarly to live axons (Figures 1D,E).

We next investigated the FRET index of the tension sensor at OL processes contacting each fiber group. The previously reported tension sensor has two fluorophores (mTFP1 and mVenus) with a tension sensor module comprising a 40-amino-acid-long elastic domain between them (Grashoff et al., 2010). The mechanical force loading on this sensor changes the FRET efficiency, because FRET can monitor the protein-protein interaction of two fluorophores. In this study, the two fluorophores (mTFP1 and mVenus) in the tension sensor were changed to the FRET pair of monomeric super-folder GFP (msfGFP)-ShadowG (Murakoshi et al., 2015). To quantitatively monitor the FRET index, we used fluorescence lifetime imaging microscopy (FLIM), which measures fluorescence lifetime changes of the donor fluorophore (Yasuda, 2006). The fluorescent proteins pair for the FLIM-FRET was msfGFP as the energy donor and ShadowG as the energy acceptor (Murakoshi et al., 2015).

**FIGURE 2:**
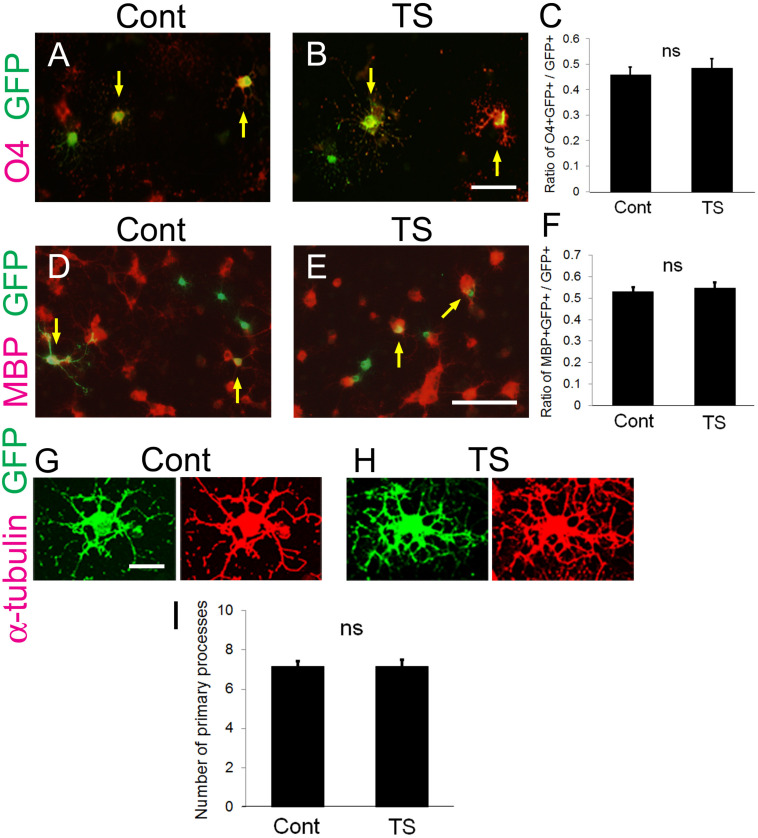
Transfection of the tension sensor does not affect OL differentiation and morphogenesis. **(A–C)** The number of O4 + immature and mature OLs [red in **(A,B)**] among the GFP + tension sensor **(TS)**-expressing cells **(B)** was comparable to that in GFP-expressing control cells **(A)** on laminin-coated dishes. The transfected cells were detected by GFP expression (green). PDGF-AA and bFGF were removed from the culture medium 1 day after transfection, and triiodothyronine (T3, 30 ng/ml) was applied. Cells were maintained for 2 additional days, and then fixed for immunostaining with anti-O4 antibody. Arrows indicate O4-GFP double-positive cells. Scale bar, 50 μm. **(C)** The ratio of O4 + GFP + cells/GFP + cells was quantified (ns, no significant difference compared with control values, Student’s *t*-test; n = 25 fields of view analyzed per condition, from three independently prepared cultures established on different days). **(D–F)** The number of MBP + mature OLs [red in **(D,E)**] among the GFP + tension sensor (TS)-expressing cells (E) was comparable to that in GFP-expressing control cells **(D)** on laminin-coated dishes. The transfected cells were detected by GFP expression (green). Cells were maintained for 5 days in the differentiation medium. Arrows indicate MBP-GFP double-positive cells. Scale bar, 100 μm. **(F)** The ratio of MBP + GFP + cells/GFP + cells was quantified (ns, no significant difference compared with control values, Student’s *t*-test; *n* = 21 fields of view analyzed per condition, from four independently prepared cultures established on different days). **(G–I)** Transfection of the tension sensor did not affect the number of OL primary processes on laminin-coated dishes. Cells were maintained for 3 days after removal of PDGF-AA and bFGF from the culture medium. OL processes were visualized by immunostaining with an anti-α-tubulin antibody (red). Scale bar, 20 μm. **(I)** The number of primary processes per GFP + OL was quantified (ns, no significant difference compared with control values; Student’s *t*-test, *n* = 27 cells analyzed per condition, from three independently prepared cultures established on different days).

At first, to examine whether transfection of the tension sensor itself does not affect OL properties and function, OPCs cultured on laminin-coated dishes were transfected with the tension sensor, and OL differentiation and the number of OL primary processes were examined. The tension sensor-transfected OPCs were transferred to culture medium without FGF2 and PDGF-AA to induce OL differentiation for 2 days. Transfection of the tension sensor did not affect the number of O4 + cells, a marker of immature and mature OLs differentiated from OPCs (Figures 2A–C). Additionally, the tension sensor did not affect the number of MBP + cells, a marker of mature OLs, which were maintained for 5 days in the differentiation medium (Figures 2D–F). We next examined the number of OL primary processes to assess OL morphology using immunocytochemistry with anti-α-tubulin antibody. Transfection of the tension sensor did not influence the number of OL primary processes (Figures 2G–I). Taken together, these results indicate that the tension sensor itself did not influence OL morphology or differentiation from OPCs to O4 + OLs.

### Higher Tension Is Generated at OL Processes Contacting Large-Diameter Fibers

**FIGURE 3:**
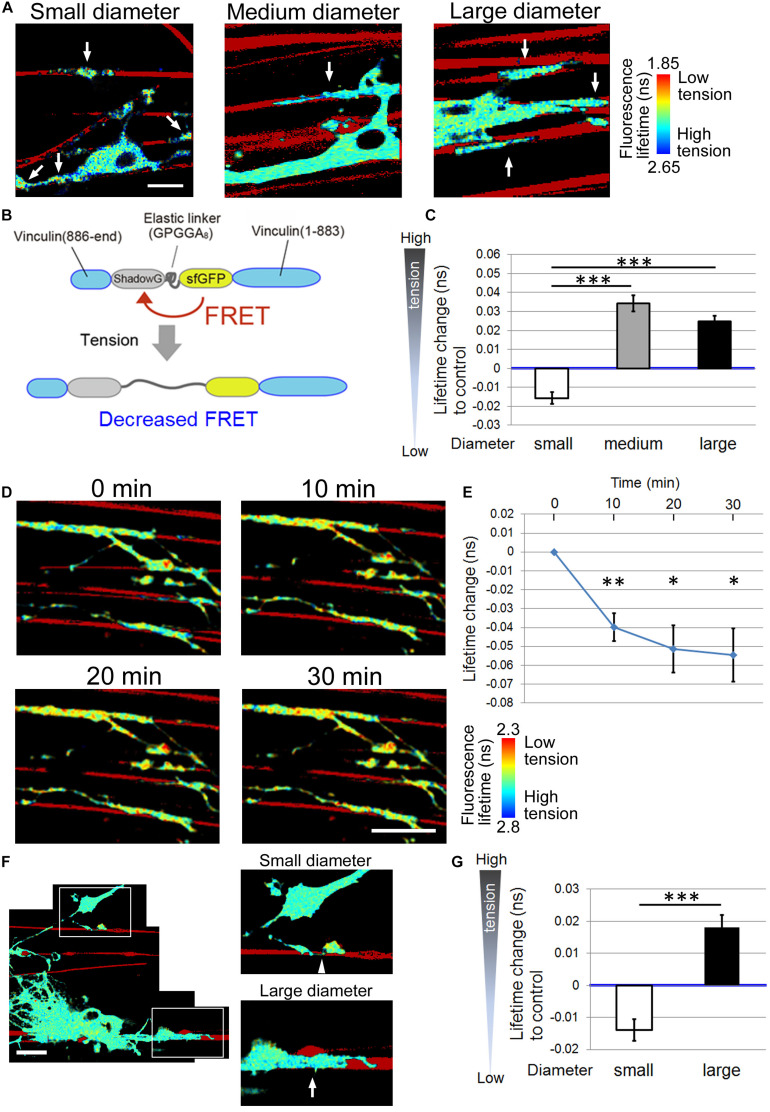
Higher tension is generated at OL processes contacting larger diameter fibers. **(A)** Representative fluorescence lifetime images of the tension sensor-expressing OL contacting nanofibers with different diameters. Sulforhodamine (red) shows polystyrene nanofibers. Arrows indicate the fluorescence lifetime images on nanofibers. Scale bar, 10 μm. **(B)** A schematic drawing of a conformational change in the elastic domain of the tension sensor. The efficiency of FRET decreases when a mechanical force is applied on it, setting the two fluorophores apart from each other. **(C)** Quantification of average fluorescence lifetime changes relative to the control value in OLs contacting small-diameter (0.55–0.9 μm), medium-diameter (1.5–1.7 μm) and large-diameter (2.5–4.0 μm) nanofibers (^∗∗∗^*P* < 0.001 by one-way ANOVA with a Tukey’s *post hoc* test; *n* = 58 areas analyzed per condition, from five independently prepared cultures established on different days). **(D)** Fluorescence lifetime images of tension sensor-expressing OLs treated with cytochalasin D (1 μM). The images show OL processes before and after (10, 20, and 30 min) cytochalasin D treatment. Sulforhodamine (red) shows polystyrene nanofibers. Cytochalasin D application to OLs significantly decreased the fluorescence lifetime, meaning lowered tension. Scale bar, 20 μm. **(E)** The time course of average fluorescence lifetime changes in response to cytochalasin D (1 μM) application (^∗∗^*P* < 0.01, ^∗^*P* < 0.05 compared with control values; Student’s *t*-test, *n* = 4 independently prepared cultures established on different days). In the cytochalasin D experiments, the gross FRET index was analyzed in each field including OL processes both with and without nanofiber contacts, and compared with the values before cytochalasin D application. In OLs treated with cytochalasin D, the average fluorescence lifetime was getting significantly shorter (indicating lower tension) within the first 10 min of application. **(F)** A representative fluorescence lifetime image of the tension sensor-expressing OL contacting both small and large nanofibers simultaneously. The tension generated by one OL on the smaller diameter fiber was lower than that on the larger diameter fiber. Sulforhodamine (red) shows polystyrene nanofibers. The right pictures are higher magnification views of the boxed areas in the left picture. The arrow and arrowhead indicate the tension sensor + signals on larger or smaller diameter fibers, respectively. Scale bar, 20 μm. **(G)** Quantification of average fluorescence lifetime changes relative to the control value in OLs contacting smaller or larger diameter nanofibers on a culture dish (^∗∗∗^*P* < 0.001 larger diameter values compared with smaller diameter values; Student’s *t*-test, *n* = 30 cells analyzed from three independently prepared cultures established on different days).

Next, we investigated the FRET index of the tension sensor at OL processes contacting small (0.55–0.9 μm), medium (1.5–1.7 μm), or large-diameter (2.5–4.0 μm) nanofibers. To quantitate the FRET index, FLIM was used to measure fluorescence lifetime changes of the donor fluorophore. The fluorescence lifetime is the time from when excitation light transits a fluorescent molecule to a high energy state, leading to the emission of a fluorescent photon. A histogram was made from the fluorescence lifetime obtained at each pixel. The average value of each histogram was then calculated, and color assignment was performed to construct a fluorescence lifetime image. FRET shortens the fluorescence lifetime of the donor fluorophore (Becker, 2012), hence it can be detected with FLIM. Using FLIM analysis, we found that OL processes contacting medium and large-diameter fibers showed longer average fluorescence lifetime, indicating higher tension, compared with small-diameter fibers (Figures 3A–C). These results suggest that higher tension is generated at OL processes contacting larger diameter axons, and physical factors influence myelin formation in response to axon caliber.

To test whether the FRET index is actually related to the mechanical force generated in OLs, the cells were treated with cytochalasin D, which depolymerizes the F-actin network and inhibits actomyosin contractility (Brown and Spudich, 1979). Cytochalasin D application to OLs significantly decreased the average fluorescence lifetime, meaning decreased tension (Figures 3D,E). This result indicates that the FRET index of the tension sensor was truly dependent on a cytoskeleton-dependent intracellularly generated force.

We further examined whether different tensions can be detected in one OL that simultaneously extends processes to both smaller and larger diameter fibers. To this end, mixed nanofibers with both smaller and larger diameters on a culture dish were prepared. We observed that the tension generated by one OL on the smaller diameter fibers was lower than that on the larger diameter fibers (Figures 3F,G), indicating that the tension difference detected by the tension sensor is not likely to be caused by the maturation state of each OL, but rather is dependent on the different fiber diameters.

### The Length of FAs Formed by OLs Positively Correlates With the Fiber Diameter

**FIGURE 4:**
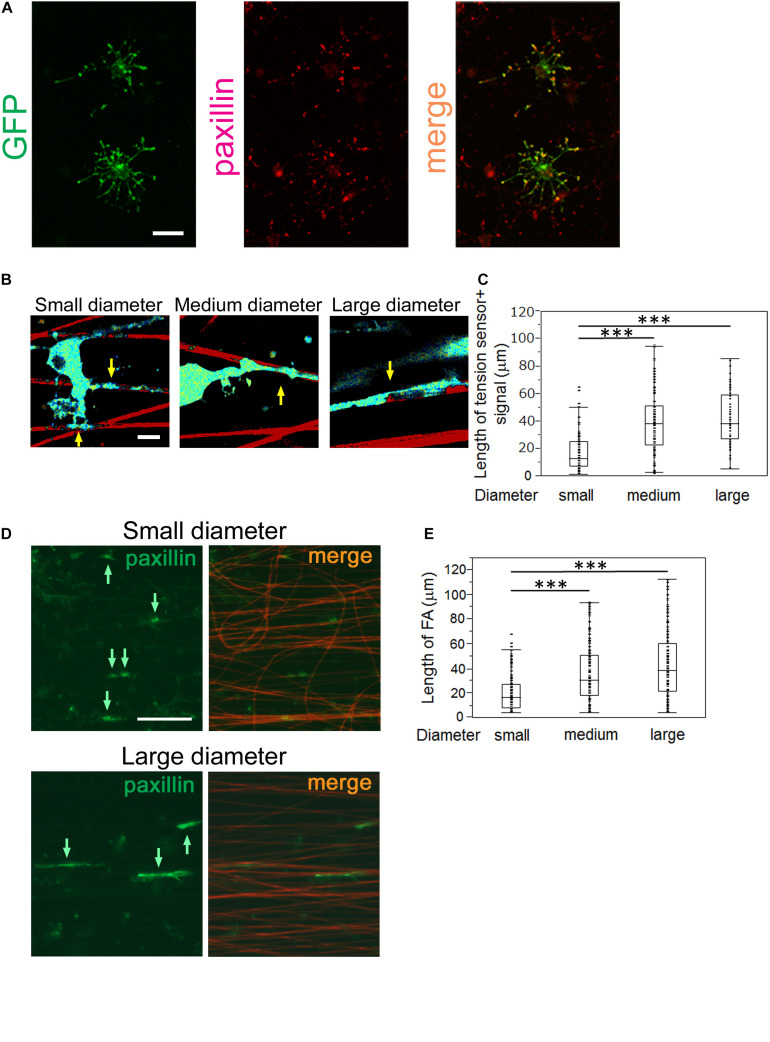
OLs form longer FAs on larger diameter fibers and shorter FAs on smaller diameter fibers. **(A)** Double immunofluorescence of GFP + tension sensor (green) and paxillin (red), a typical FA marker, is shown. The GFP + tension sensor was co-localized with paxillin at the tip of OL processes. Scale bar, 20 μm. **(B)** A representative length image of the tension sensor + signals on small, medium and large- diameter fibers. The longer signals were observed on larger diameter fibers. Sulforhodamine (red) shows polystyrene nanofibers. Arrows indicate the tension sensor + signals on nanofibers. Scale bar, 10 μm. **(C)** The length of the tension sensor + signals was quantified in each fiber group. OLs formed shorter tension sensor + signals on small-diameter fibers (0.55–0.9 μm), compared with those produced on medium (1.5–1.7 μm) and large-diameter (2.5–4.0 μm) fibers (^∗∗∗^*P* < 0.001 by one-way ANOVA with a Tukey’s *post hoc* test; n = 81 areas analyzed per condition, from five independently prepared cultures established on different days). **(D)** Immunofluorescence of paxillin (an FA marker, green) is shown. Sulforhodamine (red) shows polystyrene nanofibers. The paxillin + FAs were observed on nanofibers with small or large diameters. Arrows indicate paxillin + FAs on nanofibers. Scale bar, 100 μm. **(E)** The size of paxillin + FAs on each fiber group was quantified. The shorter FAs were formed on small-diameter fibers (0.55–0.9 μm), whereas longer FAs were produced on medium (1.5–1.7 μm) and large-diameter fibers (2.5–4.0 μm) (^∗∗∗^*P* < 0.001 by one-way ANOVA with a Tukey’s *post hoc* test; *n* = 96 FAs analyzed per condition, from three independently prepared cultures established on different days).

FA complexes are generated at the adhesion points and mechanically link the ECM to the cell, acting as key platforms for mechanotransduction. They consist of integrins, which bind to the ECM, adapter proteins, which link integrins to the cellular cytoskeleton, and cytoplasmic proteins, which are the downstream effectors of signaling pathways. A previous study has reported that the tension sensor was properly recruited to FAs where it was co-localized with paxillin, an FA protein (Grashoff et al., 2010). Hence, the tension sensor enables monitoring of the localization of FAs at OL processes. To test whether the GFP-positive tension sensor is actually localized at FAs in OLs, we performed double-immunostaining for GFP and paxillin, one of the typical FA markers. The GFP-positive tension sensor was co-localized with paxillin at the tip of OL processes on laminin-coated dishes (Figure 4A), indicating that the tension sensor was localized at FAs in OLs.

We next performed length measurements of the tension sensor-positive signals at OL processes contacting nanofibers with different diameters, which were supposed to be an indicator of FA sizes. We quantified the signal length of the tension sensor in each fiber group. OLs showed shorter tension sensor + signals on small-diameter fibers (0.55–0.9 μm), compared with those produced on medium (1.5–1.7 μm) and large-diameter (2.5–4.0 μm) fibers (Figures 4B,C). We further performed immunostaining of FAs in cultured OLs on nanofibers for 8 days. FAs on nanofibers were detected by anti-paxillin antibody and merged with sulforhodamine-positive nanofibers. FA immunostaining showed that longer FAs were formed on larger diameter fibers (Figures 4D,E), indicating that the length of FAs formed by OLs positively correlates with the fiber diameter. Previous studies have reported that FAs act as mechanotransducers that transmit various intracellular signaling pathways (Geiger et al., 2009). Among the FA components, various signaling molecules including tyrosine kinases, tyrosine phosphatases and adaptor proteins have been identified (Geiger et al., 2009). The activity of these kinases and phosphatases triggers intracellular signaling pathways that control cell properties. These previous reports and our results suggest that OLs respond to the fiber diameter and activate mechanotransduction initiated by the FAs, which might control their cytoskeletal organization and myelin formation.

**FIGURE 5:**
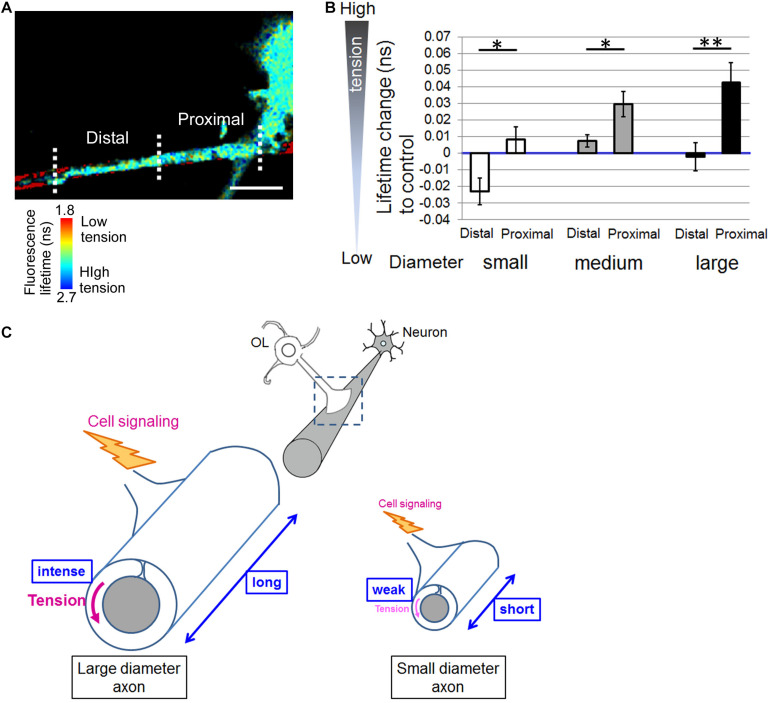
Distal contact points on nanofibers far from the OL processes exhibit lowered tension generation. **(A)** A representative fluorescence lifetime image of the tension sensor + OL processes exhibiting fiber coverage elongating unidirectionally. Images of the distal contact point and proximal contact point to the OL process are also shown. The distal contact points far from the OL processes exhibited lower tension. Sulforhodamine (red) shows polystyrene nanofibers. Scale bar, 10 μm. **(B)** Quantification of average fluorescence lifetime changes relative to the control value in the distal contact points or proximal contact points to each OL process exhibiting unidirectional fiber coverage in small-diameter (0.55–0.9 μm), medium-diameter (1.5–1.7 μm) and large-diameter (2.5–4.0 μm) groups (^∗∗^*P* < 0.01, ^∗^*P* < 0.05 proximal values compared with distal values; Student’s *t*-test, *n* = 7 areas analyzed for small-diameter fibers, *n* = 11 areas analyzed for medium-diameter fibers, *n* = 15 areas analyzed for large-diameter fibers, from four independently prepared cultures established on different days). **(C)** Schematic drawing of mechanical force generated by OLs contacting neuronal axons with different diameters. Higher tension is generated at OL processes ensheathing larger diameter axons compared with smaller diameter axons, suggesting that physical factors influence myelin formation in response to axon caliber. Additionally, OLs form longer FAs on larger diameter axons and shorter FAs on smaller diameter axons. The proximal FAs near the OL processes showed higher tension, whereas the distal FAs far from the OL processes showed lower tension. This study suggests that intracellular signaling is initiated at FAs, whose size depends on axon diameter, and controls myelin formation involving actin assembly/disassembly.

Finally, Figure 5A shows a representative image of the distal and proximal contact point to the OL process contacting nanofibers. Some OL processes exhibited unidirectional fiber coverage. We thus extracted the fiber coverage elongating unidirectionally and analyzed the FRET index in each fiber group. In the population that elongated unidirectionally, the tension sensor within the proximal contact points near the OL processes showed a longer average fluorescence lifetime, indicating high tension, whereas within the distal contact points far from the OL processes it showed a shorter average fluorescence lifetime, indicating low tension (Figures 5A,B). Previous reports have proposed a two-step model of myelination: (1) actin assembly drives OL process extension to ensheath axons, (2) local actin disassembly induces lateral spreading of the myelin membrane and its wrapping (Nawaz et al., 2015; Zuchero et al., 2015). These previous reports and our result showing lower tension within the distal contact points on nanofibers indicate that distal contact points exhibit a higher level of actin disassembly compared with proximal contact points, suggesting that more actin disassembly at the distal FAs on the axon fiber enables OL plasma membrane to lateral membrane flow for continuous myelin growth.

## Discussion

There are large and small-caliber axons in the CNS white matter. OLs ensheath various diameters of axons (Weruaga-Prieto et al., 1996). Large-diameter axons tend to be myelinated compared with small-diameter axons (Friede, 1972). Additionally, the ratio of [axon diameter] to [axon + myelin diameter] (g-ratio) is adjusted to optimum values for each axon, which is essential for exerting higher brain functions. This indicates that the axon diameter is associated with myelin formation, and there might be regulatory factors in response to the diameter. However, the mechanisms underlying axon diameter-dependent myelination have not been well clarified.

Câmara et al. (2009) have previously reported that β1 integrin is one of the factors that survey axon diameter and control myelination. They demonstrated that β1 integrin signaling is required for myelinating small-diameter axons. Integrin forms a signaling complex to initiate myelination by signal amplification. This signal amplification is necessary for triggering myelination of small-diameter axons, whereas large-diameter axons can be myelinated regardless of this amplification (Câmara et al., 2009). The axonal signal proportional to the diameter and above a certain threshold is required to initiate myelination. Integrin is one of the major FA proteins. FA is a central “hub” that transduces mechanically induced signaling from the ECM to the actin cytoskeleton. Expression of dominant-negative β1 integrin reduces this mechanical signaling, so that the signal initiated by some small axons will not be above the required threshold for myelination (Câmara et al., 2009). In the present study, we found that OLs formed shorter FAs on small-diameter axons (Figure 4), thereby providing less signals that were not above the threshold level. This is consistent with the fact that small-diameter axons in the CNS are unmyelinated in many cases. Furthermore, Suzuki et al. (2012) have reported that myelination of small-diameter axons was significantly affected in *teneurin-4*-deficient mice and that Teneurin-4 regulates integrin β1-FAK signaling. By contrast, OLs form longer FAs on large-diameter axons, which might generate signals at a level significantly higher than the threshold level, and thus not be canceled by a partial reduction in β1 integrin signaling by its dominant negative form.

Because the FA protein, integrin, has been reported to play important roles in OL-neuron interactions that regulate axon diameter-dependent myelination as mentioned above, we focused on mechanical forces generated at OL processes contacting axon fibers with different diameters. We found that large-diameter fibers induced a lower FRET index in OLs, indicating high tension, compared with small-diameter fibers (Figure 3). These results indicate that higher tension is generated at OL processes contacting larger diameter axons. We further examined whether different tensions can be detected in one OL that extends processes that contact both smaller and larger diameter fibers simultaneously (Figures 3F,G). The results suggest that the tension difference detected by the tension sensor is not caused by the maturation state of each OL. In the process of myelin formation, OLs must first extend their processes to ensheath axons, which is driven by actin assembly. When OL processes contact neuronal axons, they form FAs at the contact foci. The larger the axon diameter is, the more the tip of OL processes must expand to surround it, therefore larger FA formation is required. When mechanical forces are loaded on FAs, the FAs connecting to the actin cytoskeleton are enlarged and thickened (Geiger et al., 2009). Taken together, larger FA formation at OL processes, which involves increased generation of mechanical forces, is recruited for larger diameter axons.

A previous study has reported that the tension sensor was properly recruited to FAs where the tension sensor was co-localized with paxillin, an FA protein (Grashoff et al., 2010). We confirmed that the GFP-positive tension sensor was co-localized with paxillin in OLs (Figure 4). Hence, the tension sensor is supposed to be an indicator of the localization of FAs at OL processes. Furthermore, in the previous report, mechanical force was measured in cells transiently expressing vinculin-GFP, which was specifically localized at FAs (Balaban et al., 2001). The expression of a vinculin-GFP fusion protein enables the visualization of individual adhesion sites in live cells and the quantification of their applied force by combination with the elasticity theory (Balaban et al., 2001). Moreover, fluorescent-tagged FA proteins, such as paxillin-GFP fusion protein or zyxin-GFP, was used to monitor adhesion turnover in murine embryonic fibroblasts (Webb et al., 2004). Webb et al. (2004) have also shown that localization of zyxin-GFP to the dynamic adhesion points was due to inherent properties of the molecule. These reports demonstrated that fluorescent-tagged FA proteins can be used for monitoring FA properties and dynamics.

FAs act as a central “hub” that transduces various mechanical signaling pathways (Geiger et al., 2009). Several tyrosine phosphorylated proteins are activated in the transduction of FA-induced mechanical signaling pathways. For example, FAK activation is positively controlled by actomyosin activity and leads to upregulation of Src family kinases (Webb et al., 2004). FAK and Src family kinases are involved in myelination during its initial stages and OL morphogenesis, respectively (Forrest et al., 2009; Gonsior et al., 2014). We performed length measurements of tension sensor + signals on nanofibers and FA immunostaining with anti-paxillin antibody. Our results showed that longer FAs were formed on larger diameter fibers, while shorter FAs were formed on smaller diameter fibers, indicating the length of FAs positively correlates with the fiber diameter (Figure 4). These previous reports and our results suggest that OLs respond to fiber diameter and activate mechanotransduction initiated at FAs, which controls the cytoskeletal organization of OLs and thus myelin formation. The linkage between the actin cytoskeleton and the ECM is strengthened when force is applied to this linkage. Previous studies have reported that the size of FAs is tightly linked to the intensity of these applied forces (Balaban et al., 2001; Rape et al., 2011). Forces loaded on the actin cytoskeleton-adhesion complexes facilitate maturation of FAs in a positive feedback fashion. Maturation of FAs further activates intracellular signaling initiated at FAs (Seo et al., 2011; Gautrot et al., 2014). These previous reports and our data showing the linear dependence between mechanical force and the area of FAs in OLs indicate that larger FAs formed on larger diameter axons can facilitate more mechanical signals, such as FAK phosphorylation, compared with those on smaller diameter axons.

Our study showed that the tension sensor within proximal contact points on nanofibers near the OL processes exhibited longer fluorescence lifetime, indicating high tension, whereas within distal contact points far from the OL processes it exhibited shorter fluorescence lifetime, indicating low tension (Figure 5). Previous reports have proposed a two-step model of myelination. First, OL processes are extended to ensheath axons driven by actin assembly. Second, disassembly of actin filaments initiates membrane growth of OLs (Nawaz et al., 2015; Zuchero et al., 2015). The previous reports and our results indicate that distal contact points exhibit higher level of actin disassembly compared with proximal contact points. It is possible that intracellular cytoplasmic pressure can easily push the membrane forward at distal contact points, enabling lateral membrane flow and myelin wrapping.

## Conclusion

We observed OL-generating forces during myelination in a manner dependent on fiber diameter using a FRET-based tension sensor. Higher tension was generated at OL processes contacting larger diameter fibers compared with smaller diameter fibers. Additionally, OLs formed longer FAs on larger diameter fibers, compared with shorter FAs on smaller diameter fibers. The proximal FAs near the OL processes showed higher tension, while the distal FAs far from the OL processes showed lower tension. These results suggest a novel and interesting idea that physical factors are involved in myelin formation in response to axon diameter. The present study suggests that intracellular signaling is initiated at FAs, whose size depends on axon diameter, which control actin assembly/disassembly and thus myelin formation.

## Data Availability Statement

The original contributions presented in the study are included in the article/supplementary material, further inquiries can be directed to the corresponding author/s.

## Ethics Statement

The animal study was reviewed and approved by the Committee of Animal Experimentation of Nagoya City University Medical School and were conducted in accordance with the animal care guidelines of Nagoya City University.

## Author Contributions

TS, HMu, and HH designed the experiments. TS and HMu performed the experiments. TS and HMu analyzed the data. HMa and KI provided the materials. TS and HH wrote the manuscript. SU, AI, and NT advised the experimental processes. AH contributed to the revise experiments. All authors contributed to the article and approved the submitted version.

## Conflict of Interest

The authors declare that the research was conducted in the absence of any commercial or financial relationships that could be construed as a potential conflict of interest.

## Publisher’s Note

All claims expressed in this article are solely those of the authors and do not necessarily represent those of their affiliated organizations, or those of the publisher, the editors and the reviewers. Any product that may be evaluated in this article, or claim that may be made by its manufacturer, is not guaranteed or endorsed by the publisher.
